# Identifying factors that affect the use of health information technology in the treatment and management of hypertension

**DOI:** 10.1186/s12911-023-02284-3

**Published:** 2023-10-23

**Authors:** Aysan Faezi, Hadi lotfnezhad Afshar, Bahlol Rahimi

**Affiliations:** 1 Health and Biomedical Informatics Research Center, Urmia University of Medical Sciences, Urmia, Iran; 2 Department of Health Information Technology, School of Allied Medical Sciences, Urmia University of Medical Sciences, Urmia, Iran

**Keywords:** Health information technology, Hypertension, Hypertension management, Patients management, Blood pressure

## Abstract

**Background:**

We conducted this study with the aim of identifying factors that affect the use of health information technology in the treatment and management of hypertension.

**Methods:**

This paper is a descriptive-analytic study conducted in 2022. To obtain relevant articles, databases including Scopus, Web of Science, IEEE, and PubMed were searched and the time period was between 2013 and 2022. Based on the review of similar articles, a five-point Likert scale checklist was developed in the second phase. The statistical population of the present study was specialist physicians (N = 40) and patients (N = 384). In order to analyze the data, SPSS Statistics 24 was used. To analyze the data obtained from the checklist, we used summary statistics (mean and standard deviation).

**Results:**

As a result of the review literature process, 50 papers were screened, that based we can distinguish motivational and inhibitory factors affecting the use of health information technology in hypertension management. Indeed, Motivational factors and inhibitory factors can be classified into five groups: organizational, economic, technical, personal, and legal/moral factors. Based on the results of the checklist, the factors that were identified as most influential on motivation and inhibitory patients and specialist physicians’ to use of health information technology to manage and treat hypertension.

**Conclusion:**

Utilizing technologies for hypertension, its management can be improved by identifying motivating and inhibiting factors. Our approach can improve the acceptability of these technologies, save costs, reduce long-term complications of hypertension, and improve patient quality of life.

**Supplementary Information:**

The online version contains supplementary material available at 10.1186/s12911-023-02284-3.

## Introduction

Hypertension, or high blood pressure (BP), is a chronic disease that causes high blood pressure in the arteries [1]. This disease accounts for approximately 13% of all deaths and is known as the “Silent Killer” [2]. According to World Health Organization (WHO) official statistics, more than 1.28 billion adults aged 30–79 are hypertensive worldwide, with two-thirds living in low- and middle-income countries. In addition, 46% of adults with hypertension are unaware of their condition [3]. It is estimated that less than half of adults (42%) who have hypertension are identified and treated, while about one out of five (21%) have hypertension under control [3].

The challenge of controlling hypertension remains largely unmet for public health systems [4]. In spite of advances in blood pressure measurement techniques, antihypertensive drugs that are both effective and safe, and various health information technology systems a large number of hypertensive patients are still not properly was identifying. In addition, a significant number of those receiving antihypertensive treatment fail to achieve satisfactory blood pressure contro [4, 5]. As a result, comes as no surprise that hypertension contributes to disease burden and disability worldwide, even in developing nations [4, 6]. Hypertension, like other chronic diseases, has significant negative effects on the affected people and society from an economic, psychological, and social perspective [1]. Hypertensive patients face a variety of daily challenges as a consequence of the complications associated with this disease [4].

The use of health information technology systems is expected to help patients with chronic diseases improve their quality of life by increasing their awareness of the diseases they have [7–9]. Indeed, raising awareness and providing continuous education can help treat these patients more effectively [10–12]. In the meantime, health information technologies can play an important role due to having made it easy to access information [13–15]. Until today, many studies have focused on the design and development of health information tools and systems for the management, prevention, or treatment of various diseases [10–12, 16].

In order to prevent, manage, and treat hypertension as a chronic disease, many scientists around the world have designed and developed health information systems [16, 17]. When evaluating these health information systems, researchers examine what makes stakeholders use or not use them [18, 19]. Identifying these factors for scientists is important because greater detail will allow for providing better studies and, better design of health information technology systems [19, 20]. As a result, the better designs of these systems will be accepted by a higher percentage and will be more user-friendly from the point of view of users (Patients). It should be borne in mind that users’ persuasion to utilize health information systems is one of the most important goals of researchers and builders of these systems [7, 21, 22]. For this purpose, this study identifies factors that affect the use of health information technology in the treatment and management of hypertension.

## Methods

In 2022, we conducted this study in two main phases as a descriptive-analytic study.

### First phases

The first phase was an analysis of the literature in order to identify factors that are supposed to affect the use of health information technology in hypertension management and treatment. This phase partially follows the PRISMA-ScR checklist [23]. To begin, we searched the following keywords in PubMed, Scopus, IEEE, and Web of Science databases:

**A**. ‘‘Blood Pressure” OR ‘‘Hypertension”

**B**. ‘‘Self-care” OR ‘‘Self-management” OR ‘‘Self-care Strategies” OR ‘‘Self-management Strategies” OR “Treatment”

**C**. ‘‘Medical Informatics” OR ‘‘Health Informatics” OR ‘‘Health Information Technologies” OR ‘‘Clinical Informatics” OR ‘‘m-Health” OR “e-Health” OR “Healthcare”

**E**. [A] AND [B]

**F**. [A] AND [C]

**G**. [A] AND [D]

**H**. [A] AND [B] AND [C]

Review and research articles (full-text access) in the English language were considered for inclusion. They had to be published between 2013 and 2022 and address the issue of the effect of health information technology on hypertension treatment and management. Abstracts, short reports, letters to the editor, and systematic review protocols were not included. Also excluded were documents that did not reference a human subject or that discussed the use of health information technology in the treatment and management of hypertension. We manually searched Google Scholar and PubMed to identify additional relevant studies. Furthermore, we reviewed all sources cited in the articles selected for the study to make the search even more thorough and highly reliable.

### Second phases

In second phase, based on the review of similar articles, a five-point Likert scale checklist was developed in the second phase. The population of the present study was composed of two groups: the first group included obstetricians, cardiologists, and nephrologists in Urmia Medical Sciences Teaching Hospital (West Azerbaijan Province, Iran) and the second group included all patients with hypertension who had visited the aforementioned centers. Due to the small number of specialist physicians (N = 40), all of them were selected, and due to the unlimited number of patients based on determining sample size Krejcie, R. V., & Morgan [24], we selected the population of patients (N = 384). Participants were provided with the link (URL) to the checklist in Google Forum. The criteria we used to select participants were computer literacy (ability to use smartphones, laptops, social networks, and internet search) and technology use. To analyze data, we used SPSS Statistics 24 (IBM Corp, Armonk, NY, USA). To analyze the data obtained from the checklist, we used summary statistics (mean and standard deviation). In addition, we use linear regression. An effective way to model the relationship between a scalar response and an explanatory variable is to use linear regression [25].

## Result

### Analysis of the review literature

**Fig. 1:**
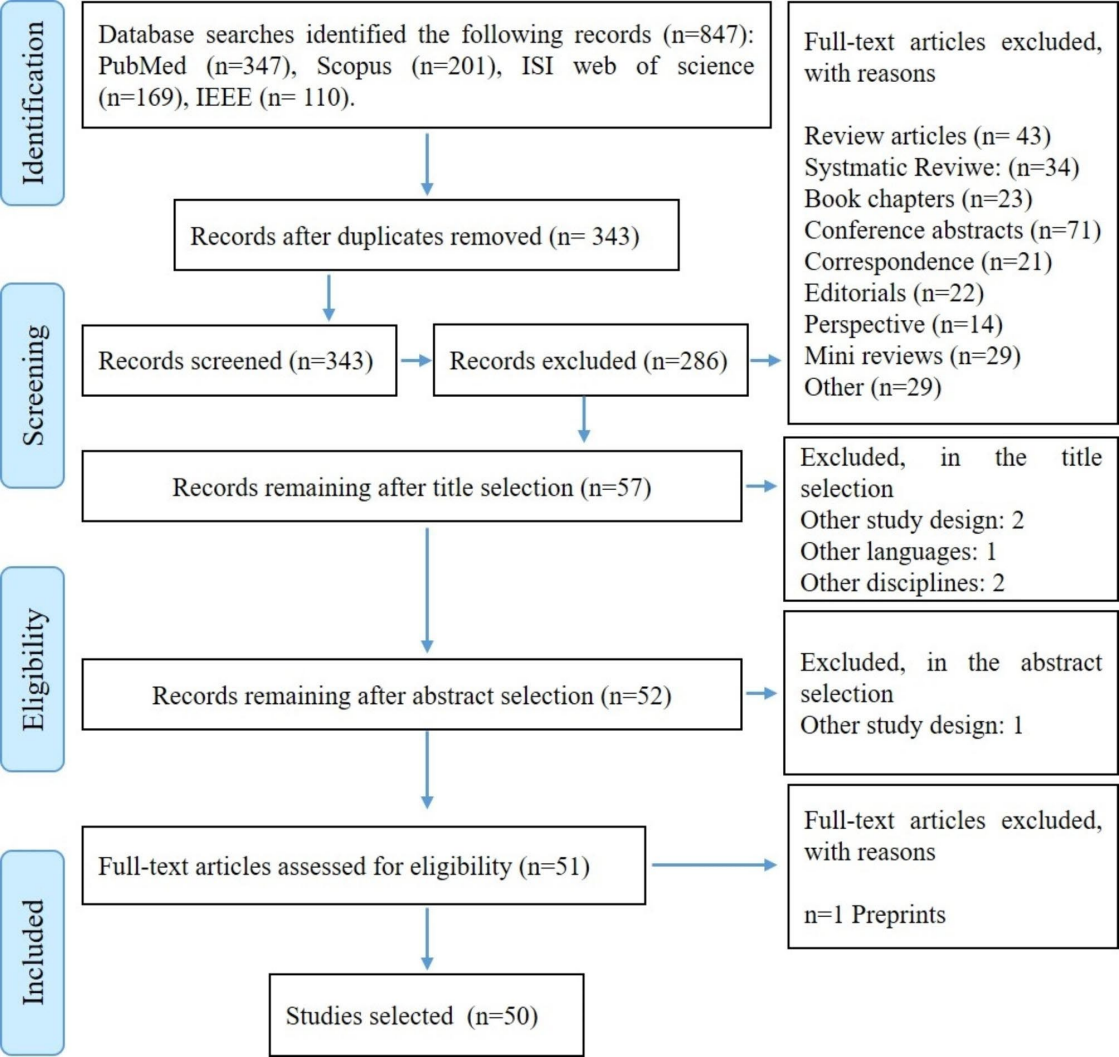
Summary of results of search and screening of the studies

The PRISMA flow diagram shows that 827 potentially relevant articles were identified from the initial search. Considering the exclusion criteria of 484 articles, they were removed after reviewing their titles and abstracts. To determine whether the remaining 343 articles met the inclusion criteria, was read the full text of each article. It should be noted that we did not find any additional articles in the references to eligible studies (Fig. 1). As a result of the screening process, 50 articles were selected (Appendix 1).

### Identified factors

Based on the results of the literature review, we can distinguish motivational and inhibitory factors affecting the use of health information technology in hypertension management. Results showed that 40 articles included both motivational and inhibitory factors, 9 articles included only motivational factors, and one article included only inhibitory factors (Appendix 2). Motivational factors in the use of health information technology have been highlighted in 49 studies. Also, inhibitory factors in the use of health information technology have been highlighted in 41 studies. Indeed, motivational factors and inhibitory factors can be classified into five groups: organizational, economic, technical, personal, and legal/moral factors (Appendix 3).

### Evaluating factors and groups

The article review was conducted in order to develop a five-point Likert scale checklist. The checklist had two categories: Motivational factors (N = 30) and Inhibitory factors (N = 24) in the 54 items, which were divided into five groups: Economic (N = 5), Personal (N = 24), Organizational (N = 12), Technical (N = 10), and Legal/Moral (N = 3).

### Demographic of participants

According to the statistical population community of 384 patients, we were able to communicate with 324 of them. Finally, 312 questionnaires were collected. The collecting questionnaires among specialist physicians were in person and were conducted by a researcher from our team (AF). Table 1 shows the demographic characteristics of patients and specialist physicians (Table 1).

**Table 1:** Demographic characteristics of participants

Participants			N
Patients	Gender	Men	124
		Women	176
	Age (years)	20–30	15
		31–40	32
		41–50	84
		51–60	181
	Duration of affected hypertension (years)	1–4	171
		5–9	81
		10–14	46
		Over 15	14
Specialist physicians	Gender	Men	15
		Women	25
	Specialty	Cardiologist	8
		Obstetricians	19
		Nephrology	13

### Motivational factors

Based on the results of the checklist, the following factors have been found to be most influential on patient and specialist physicians’ motivation to use health information technology to manage and treat hypertension. From the point of view of patients: in the economic factor, the reduction of treatment costs with an average of (3.18); in the individual factor, the highest average is related to the increase in treatment follow-up (3.77); in the organizational factor, the highest average is related to making technology available (3.84); in the technical factor, the highest average is related to the possibility of easy data sharing (3.90); and in the legal and moral factors, the highest average is related to the anonymity of the patient’s identity in a disturbing situation (3.41). According to specialist physicians: in the economic factor, the highest average is related to economic support (3.57); in the individual factor, the highest average is related to the increase in treatment follow-up (3.65); in the organizational factor, the highest average is related to the improvement of service quality (3.60); in the technical factor, the highest average providing voice reminders and alerts (3.40); in the legal and moral factor is also the average of anonymity of the patient’s identity in a disturbing situation (3.45) (Table 2).

**Table 2:** The mean and standard deviation between views of patients and physicians about using health information technology in hypertension management (Motivational factors)

Motivational factors		**Patients**		**Specialist physicians**	
Groups	Factors	Mean	SD	Mean	SD
Economic	Reduce the cost of treatment	3.18	1.03028	3.47	0.84694
	Economic support	2.83	1.09022	3.57	0.95776
	Providing free services to patients	3.03	0.96154	3.30	0.88289
Personal	Increase treatment follow-up	3.77	1.30675	3.65	0.62224
	Familiarity with new treatment methods	2.62	1.10886	3.45	0.74936
	Responding to patient information needs	3.12	1.26134	3.52	0.90547
	Reducing blood pressure complications in the long term	3.20	1.15714	2.77	1.12061
	Promoting patient self-management	3.36	1.16323	3.15	0.92126
	Saving time	3.60	1.30132	3.50	1.37747
	Long duration of illness	2.74	1.09362	3.57	0.85011
	Fast learning to use technologies	3.12	1.19705	3.42	0.87376
	High responsibility and dynamism of the patient	3.24	1.06684	3.52	0.84694
Organizational	Making technology available	3.13	1.21742	3.23	1.03775
	Advising the doctor to the patient to use these technologies	3.26	1.18559	3.42	0.78078
	Promotion about technology	2.98	1.19620	3.07	1.14102
	Training and introduction of new technologies before use	3.78	1.23125	3.15	1.02657
	Government support	3.09	1.17150	3.30	0.79097
	Training and introduction of new technologies before use	3.83	1.15623	3.25	0.80861
	Cooperation between the service provider and the patient with the technology manufacturer	2.77	1.17904	3.50	1.03775
	Increasing access to health services	2.77	1.18211	3.40	0.98189
	Providing equitable health services	2.44	1.10088	3.60	0.84124
	Social and family support	3.15	1.10769	3.22	0.91952
Technical	User-friendly design	3.51	1.26694	3.22	0.65974
	Up-to-date information provided	2.74	1.14813	3.22	0.91952
	Verification and verification of information provided by experts	2.89	1.03385	3.27	0.71567
	Providing voice reminders and alerts	3.46	1.03220	3.40	0.90014
	Easy sharing	3.90	1.13910	3.30	0.79097
Legal/Moral	Anonymity of the patient’s identity	3.41	1.19916	3.45	0.59700

All five groups (organizational, economic, technical, personal, and legal/moral) of motivational factors significantly predicted hypertension management scores based on patients’ and physicians’ views. However, the economic (R2 = 0/681) and legal/moral (R2 = 0/651) groups had the highest effects in predicting hypertension management scores based on patients’ and physicians’ views respectively (Table 3).

**Table 3:** Results of the multiple linear regression about patients and physicians motivational groups

Motivational groups	Patients					Specialist physicians				
	P-value	Multiple R	R square	Adjusted R square	Standard error	P-value	Multiple R	R square	Adjusted R square	Standard error
Economic	0/001	0/694	0/681	0/666	0/6580	0/001	0/315	0/300	0/268	0/6354
Personal	0/001	0/626	0/591	0/577	0/6358	0/001	0/314	0/291	0/277	0/3698
Organizational	0/001	0/446	0/417	0/407	0/6587	0/001	0/520	0/470	0/452	0/6598
Technical	0/001	0/548	0/510	0/497	0/6932	0/001	0/412	0/388	0/367	0/4785
Legal/Moral	0/001	0/462	0/452	0/432	0/6742	0/001	0/699	0/651	0/633	0/3698

### Inhibitory factors

Based on the results of the checklist, the following factors have been found to be most influential on patient and specialist physicians’ inhibitory to the use of health information technology to manage and treat hypertension. According to the patients: in the economic factor, the highest average is related to the high cost of setting up technologies (3.31), in the individual factor, the highest average is related to low education (3.77), in the organizational factor, the highest average is related to the lack of infrastructure Suitable (3.27), in the technical barrier factor, the highest average is related to the problem of accessing the Internet and mobile phone (3.48), and in the legal and ethical factor, the highest average is related to privacy concerns (3.73). According to specialist physicians: in the economic factor, the highest average is related to the high cost of setting up technologies (3.35), in the individual factor, the highest average is related to resistance to change (3.95), in the organizational factor, the highest average is related to the lack of policy and long-term plans (3.47), in the dimension of the technical factor, the highest average is related to the problem of accessing the Internet and mobile phone (3.62), in the legal and ethical factor, the highest average is related to the increase in medical and legal responsibility (3.75) (Table 4).

**Table 4:** The mean and standard deviation between views of patients and physicians about using health information technology in hypertension management (Inhibitory factors)

Inhibitory factors		Patients		Specialist physicians	
Groups	Factors	Mean	SD	Mean	SD
Economic	Bad economic conditions of users	3.23	1.03348	3.48	0.68333
	High cost of setting up technology	3.31	1.04209	3.35	0.66216
Personal	Low education	3.77	1.10368	3.60	0.70892
	Old age	3.61	1.25799	3.37	0.66747
	Low knowledge and awareness	3.37	1.14664	3.45	0.71432
	Lack of technology literacy	3.13	1.06735	3.75	0.86972
	Existence of physical problems	3.30	1.19594	2.75	0.95407
	Desire for face-to-face communication between doctor and patient	3.53	1.20693	3.38	0.67338
	Limited insight into the disease	3.09	1.13008	3.60	1.00766
	Geographical location	3.13	1.09340	3.55	0.81492
	The experience is a reflection of the previous experience	3.05	1.10316	3.22	0.99968
	Increased patient stress	2.81	1.18786	3.10	1.19400
	Lack of confidence in the presented content and self-monitoring	3.30	0.92265	3.65	0.83359
	Ethnic and cultural problems	3.01	0.95514	3.20	1.15913
	Resistance to change	2.44	1.23771	3.95	0.81492
Organizational	Lack of proper infrastructure	3.27	0.86860	2.97	0.89120
	Lack of policy and long-term plans	3.21	0.91938	3.47	0.87669
Technical	Incomprehensibility of technologies	2.94	0.95109	3.10	0.87119
	Lack of training in the use of technologies	2.90	1.04677	3.10	1.00766
	Technical problems of technologies	2.90	1.05959	3.25	0.83972
	A large volume of content and information that causes confusion	2.92	1.08763	3.15	0.89299
	Internet and mobile phone access problem	3.48	1.73274	3.62	0.77418
Legal/Moral	Privacy concerns	3.73	1.04719	3.50	0.90582
	Concern for security	3.72	1.15094	3.79	0.76707

All five (organizational, economic, technical, personal, and legal/moral) of inhibitory factors significantly predicted hypertension management scores based on patients’ and physicians’ views. However, the personal (R2 = 0/855) and organizational (R2 = 0/200) groups had the highest effects in predicting hypertension management scores based on patients’ and physicians’ views respectively (Table 5).

**Table 5:** Results of the multiple linear regression about patients and physicians inhibitory groups

Inhibitory groups	Patients					Specialist physicians				
	P-value	Multiple R	R square	Adjusted R square	Standard error	P-value	Multiple R	R square	Adjusted R square	Standard error
Economic	0/000	0/714	0/700	0/689	0/6071	0/003	0/114	0/109	0/102	0/3121
Personal	0/000	0/865	0/855	0/842	0/6557	0/003	0/107	0/104	0/100	0/2561
Organizational	0/000	0/520	0/499	0/490	0/6400	0/000	0/209	0/200	0/175	0/3651
Technical	0/000	0/710	0/697	0/685	0/5575	0/001	0/124	0/116	0/111	0/3652
Legal/Moral	0/000	0/477	0/465	0/454	0/5365	0/001	0/157	0/147	0/135	0/4569

## Discussion

### Principal findings

This study aimed to identify factors that affect the use of health information technology in the treatment and management of hypertension. To this end, we first analyzed the literature to identify factors that affect the use of health information technology in hypertension management and treatment, and next with the help of specialist physicians and patients, we identified these factors. As a result of the literature review, we could distinguish motivational and inhibitory factors affecting the use of health information technology in hypertension management; they can be classified into five groups: organizational, economic, technical, personal, and legal/moral factors. A checklist was used to ask specialist physicians and patients to choose the most effective factors, as shown in Tables 2 and 4.

### Implications of motivational and inhibitory factors for Health Care Providers

Healthcare service providers often try to make technology-based products that help in the course of treatment available to patients for free, which can be effective in reducing the costs of the disease. In fact, providing free services to patients is one of the motivating factors for patients to use technology, which was the concern of patients and specialist physicians in our study. However, some technology-based products are expensive, this issue can affect the number of users [26]. In addition, the high cost of designing and developing some technology-based products can also affect the technology distribution policies of healthcare providers [18, 22].

According to our findings, treatment follow-up and time-saving were among the most important personal motivating factors for using technology-based products from the perspective of patients and specialist physicians. In fact, for reasons such as availability (accessibility) to services at any time and place (i.e., 24/7), these products cause more participation and increase the sense of responsibility of patients towards their health [27]. Definitely, regular treatment follow-up improves patients and increases the quality of service and care provided by healthcare providers [10, 27]. Among the personal inhibiting factors from the point of view of patients and specialist physicians, one can mention the low level of literacy (i.e. low education). This issue is of concern when the information provided by the technology-based product (e.g., educational app) is very specialized or set at a high scientific level; the same makes patients not motivated to use the product [10, 11]. It should be noted that information needs assessments are very important before the development of a technology-based product because it is possible to obtain the scientific level and educational desire of patients and provide information to patients [11, 12].

Organizations motivate patients to use technology by teaching them how to use it before implementing it, which allows them to become more familiar with its capabilities and facilities. It also introduces users to the benefits of using technologies, including providing fair health services to patients regardless of geography (such as a city or village access) and time (depending on natural phenomena or political conditions), increasing the level of acceptance of technology by users. One of the organizational inhibiting factors for the use of technology-based products from the patient’s point of view is the lack of appropriate technology infrastructure. But the organizational inhibiting factor from the specialist physicians’ point of view is the lack of long-term policies and plans. It should be noted that the use of technology results is as much a promising strategy for patients in the event that things like the lack of proper infrastructure such as internet problems (i.e. speed and coverage) and lack of resources and lack of policy and planning are not possible [10]. Duration is an important concern and causes a waste of time and money and increases the workload for health providers and increases the probability of failure in providing services to patients.

According to our findings, technical features including easy sharing, user-friendly design, and provision of voice reminders and warnings are among the things that motivate patients to use technology-based products; these features are also highly important in the view of specialist physicians. According to studies, the easy sharing feature can cause the sharing of useful and correct information between users, in fact, this issue can help to expand the users of technology-based products [10, 18]. User-friendly design is also a very important issue, some scholars have called it a human rights issue because poor user-friendly design discourages people from developing software [28, 29]. Providing warning features help to remind patients to schedule a doctor’s appointment or take time medications [11]. Among the most important inhibitory factors from the point of view of patients was the problem of accessing the internet and mobile phones; in fact, the speed of the internet and access to a tool that can run the desired app/software is the main requirement for using the designed product. According to studies, developing countries face this problem more due to poor technological infrastructure [10]. From the point of view of specialist physicians, technical problems of technologies are among the most important problems related to technology-based products, which are the main deterrent. It is very important that the developed product is well designed from a technical point of view and can be implemented without causing problems and creates the least feeling of confusion during use [10, 12]. Attention to it can increase usability [12, 26].

The anonymity of the patient’s identity is a feature that is the only motivating factor for the use of technology-based products from the perspective of patients. This means that the technology-based products do not retain any identifying information about the patient. Anonymity and confidentiality are essential to protect service users and carers, placement providers and mentors, as well as the assessment candidate [30, 31]. Furthermore, it is required to comply with data protection laws and good ethical principles [32]. Failure to consider this issue reduces the importance of patient privacy and security, which was one of the issues inhibiting the use of technology-based products from the point of view of specialist physicians.

### Comparison with prior work

The findings of our study are similar to the results of the Heuvel et al. [33] study, which showed that the use of technologies has a positive effect on reducing treatment costs. The results of McGillicuddy et al. [34] study, which does not consider economic and social support among the motivating factors in the use of technologies. Chérrez-Ojeda et al. [35] also listed economic problems as one of the most important factors preventing the use of technologies and pointed out that economic support is very effective for the greater use of technologies. In another study by Barsky et al. [36], which investigated the effect of mobile phone intervention in reducing hypertension, the increase in treatment follow-up was considered one of the important and influential motivational factors, such as the results of our study. In another study conducted by Citoni et al. [37], with the aim of investigating telemedicine in controlling hypertension in the context of the COVID-19 pandemic, the most important motivating factor was the availability of technologies, and the findings of our study also confirm the importance of this issue. In the technical factor, our study was similar to that of Hallberg et al. [38]. In their study, they positively express the effect of providing warnings and voice reminders and find it effective in improving individual habits. In our study, in addition to confirming it, we found the simple and user-friendly design of technologies as a positive factor. It should be noted that the design of the appropriate user interface makes the users more persuasive and this issue makes it easier for the patient to accept the designed system [10, 26, 39–41].

### Limitations and strengths

In this study, we provide an overview of the various approaches to managing hypertension using health information technology. Researchers can use the findings of the first phase to evaluate their intervention elements compared with previous approaches based on the results of the first phase. The purpose of this study was to examine the factors influencing the use of technology in the treatment and management of hypertension with the help of patients and specialists. The findings can assist designers, developers, and policymakers in the field of healthcare. The Ovid and EMBASE databases were not explored. Despite our wide-ranging search, we were able to gather perspectives from a variety of sources and countries; however, our search was not comprehensive, which limited the results. Another limitation of this study was the small number of patients included, and although we did our best, the small number created another limitation.

## Conclusion

Using health information technology to improve hypertension treatment and management is a great opportunity. In the present study, motivational and inhibitory factors were found to affect the use of health information technology in hypertension management. Each of these factors can also be classified into five categories: organizational, economic, technical, personal, and ethical/legal.

As a result of these factors, some of the major limitations of current therapeutic strategies may be prevented, such as low patient adherence to treatment, physician inertia, and poor communication between patients and providers. It is possible to save money, reduce complications of hypertension, and improve patient health by paying attention to these factors when using health information technology in hypertension management. Additionally, focusing on these factors will prevent time and resource waste in the design and implementation of technology.

### Electronic supplementary material

Below is the link to the electronic supplementary material.

Supplementary Material 1

## Data Availability

Please contact the corresponding author if you would like access to the datasets used and/or analyzed during this study.
