# Supplementary material for: Identifying factors that affect the use of health information technology in the treatment and management of hypertension
Source: BMC Med Inform Decis Mak. 2023 Oct 23;23:235. doi: 10.1186/s12911-023-02284-3 (PMC10591361; doi:10.1186/s12911-023-02284-3)
Supplement: Supplementary file 1 — Supplementary Material 1 [file 12911_2023_2284_MOESM1_ESM.docx]

**Appendix 1: Analysis of the review literature**

Table 1. The descriptive review of articles/Author(s)/Year Country/ Objective/ Methods/ Findings

| **NO** | **Author(s)/Year** | **Country** | **Objective** | **Methodes** | **Findings** |
| --- | --- | --- | --- | --- | --- |
| 1 | )[Shaw et al., 201](#_ENREF_42)3) | US | Through the implementation of an evidence-based self-management telephone intervention to facilitate hypertension management in large complex health systems, we sought to answer the following questions: What is organizational readiness to implement the intervention? What are the facilitators, barriers, and specific contextual factors that may affect organizational readiness for change? | Qualitative and quantitative | **Inhibiting factors**: increase in workload, need to redesign the work process, lack of government support and appropriate policies, lack of trust in the presented materials, time-consuming, lack of proper infrastructure, lack of resources, financial problems, concern about prolonging the installation time and Implementation and execution of the system.  **Motivating factors**: high work commitment, individual motivation, ability to work remotely, quick access to services, cost-effectiveness, high knowledge and awareness (literacy), technical support, creating a competitive environment between organizations, providing financial incentives, suitable user-oriented design, and flexible, improving coordination and cooperation between providers, cooperation between the user and the system developer, the level of readiness of organization members, organization readiness for change. |
| 2 | ([Robins et al., 2013](#_ENREF_40)) | US | Examining the challenges and opportunities of implementing a hypertension monitoring trial at home and electronic communication | Qualitative | **Inhibiting factors**: the cost of providing technologies, old age, low self-efficacy ability, financial problems, increased workload, lack of rules and regulations, and problems with repayments.  **Motivating factors**: supporting providers, saving costs, facilitating information exchange and sharing, improving the quality of care, and improving the accuracy of technologies. |
| 3 | ([Hanley et al., 2013](#_ENREF_20)) | Scotland | Examining the experiences of patients and professionals participating in a randomized controlled trial (RCT) of remote blood pressure (BP) monitoring with primary care support and identifying factors facilitating or hindering the effectiveness of the intervention. | Qualitative | **Motivating factors**: immediate and fast reading, convenient sharing of information, helping the treatment process, reducing face-to-face visits, and improving patient-provider communication.  **Inhibiting factors**: anxiety and fear of technology, lack of motivation, lack of integration of technologies with the work process, and increase in workload. |
| 4 | ([Flynn et al., 2013](#_ENREF_14)) | US | Examining the perspectives of patients and their family members regarding facilitators and barriers to self-management of hypertension in urban African Americans. | Qualitative | **Motivating factors**: support from family members, improving effective patient communication with service providers, follow-up treatment after appointments.  **Inhibiting** **factors**: lack of access to resources, insufficient awareness, limited health knowledge, lack of motivation. |
| 5 | ([Patel et al., 2013](#_ENREF_36)) | US | Assessment of antihypertensive medication adherence with an automated medication reminder app for personal mobile phones in patients. | RCT | The overall result indicated that the mobile phone-based automatic medication reminder system is promising in improving medication adherence and hypertension in people at cardiovascular risk. |
| 6 | ([McGillicuddy et al., 2013](#_ENREF_28)) | US | To assess the attitudes of renal transplant recipients toward mobile phone-based remote monitoring and management of their medical regimen, and to identify demographic or clinical characteristics that impact on this attitude. | Qualitative and quantitative | **Motivational** **factors**: being free, affordable, increasing quality of life, increasing self-efficacy, positive attitude, adherence to medication.  **Inhibiting** **factors**: age, annual income, education level, economic and social support, cost of technologies, lack of interest, privacy concerns. |
| 7 | ([Lee et al., 2014](#_ENREF_26)) | Australia | Our goal was to explore the navigational needs of consumers with chronic health conditions in finding online health information within the broader context of consumers’ online health information-seeking behaviors. Potential barriers to online navigation were also identified. | Qualitative | **Motivating** **factors**: suitable user-oriented and flexible design, user-friendliness, confidence in personal abilities, self-efficacy, sufficient computer skills, provider cooperation and support, quick access, up-to-date information, high knowledge and awareness (literacy).  **Inhibiting** **factors**: high volume of information, lack of trust in the presented materials, lack of motivation, heterogeneous information. |
| 8 | ([Fiksdal et al., 2014](#_ENREF_13)) | US | We conducted this study to gain a deeper understanding of online health-searching behavior in order to inform future developments of personalizing information searching and content delivery. | Qualitative | **Motivating** **factors**: motivation, the anonymity of a person's identity in annoying situations, saving time and money, more awareness, increasing patient dynamism, facilitating communication between the patient and the provider, computer literacy, ease of use, access to up-to-date and accurate information, User-friendly design, reliability and assurance of information accuracy, simplification and personalization of database information, treatment follow-up after the appointment. |
| 9 | ([Hall et al., 2014](#_ENREF_18)) | US | Explores patients' perceptions and the current use of technology for managing HF symptoms (MHFS). | Qualitative | **Motivating** **factors**: social and family support, government support, providing reminders, scheduling appointments, having a daily schedule, reducing costs, saving time, quick access, better patient-provider communication, ease of use, adequate training, appropriate design, and integration of technologies. Validation of technologies.  **Inhibiting** **factors**: fear of technology, unwillingness, low self-efficacy, lack of trust, economic problems, low health literacy, old age. |
| 10 | ([Legido-Quigley et al., 2015](#_ENREF_27)) | Colombia | Survey of knowledge, attitude, behavior and health care experiences of patients regarding prevention, diagnosis, management and control of hypertension in Colombia. | Qualitative | **Motivating** **factors**: the level of patient knowledge and awareness, the level of patient experience, patient self-efficacy, patients' attitude, providing free services, better communication between the patient and the provider, quick access, providing quality services, making appointments, saving money and time, providing support providers, increasing accountability.  **Inhibiting** **factors**: lack of access to technologies, lack of providing sufficient information, interest in face-to-face consultations, lack of organizational culture, lack of trust in information, economic limitations, the severity of the disease |
| 11 | ([Glynn et al., 2015](#_ENREF_16)). | Ierland | Explore patients’ views and experiences of using technology-based self-management tools for the treatment of hypertension in the community. | Qualitative | **Motivational** **factors**: level of medical knowledge and awareness, self-efficacy, responsibility, easy access, reducing stress, improving treatment quality, increasing patient participation, increasing patient knowledge, and awareness, having sufficient motivation, helping to improve personal habits, improving communication between the patient and Provider, access to up-to-date information.  **Inhibiting** **factors**: lack of trust, interest in face-to-face visits, the accuracy of the information, data security, availability of technologies. |
| 12 | ([Hallberg et al., 2016](#_ENREF_19)). | Sweden | Explore patients’ experiences of an interactive mobile phone-based system designed to support the self-management of hypertension. | Qualitative/RCT | **Motivating** **factors**: simplicity, ease of use, providing warnings and reminders, helping to change individual habits, increasing the motivation to adhere to treatment, perceived usefulness, increasing participation in care, responsibility, increasing awareness, designing according to the needs of users, social support, economy Saving costs.  **Inhibiting** **factors**: technical problems, internet problems, lack of sufficient training, lack of technical support, lack of proper design of system output, lack of motivation, and economic problems. |
| 13 | ([Ganapathy et al., 2016](#_ENREF_15)) | UK | Assess suitability of remote self-monitoring of blood pressure with an innovative use of technology in detecting raised blood pressure in pregnancy. | RCT | This technology provides accurate information and visual cues including safe remote transmission instantly. 90% of women agreed that the kit was easy to use and 78% preferred this model of testing at home. |
| 14 | ([Chung et al., 2016](#_ENREF_9)) | US | Evaluate BP kiosk acceptability and usability, as well as its effects on the workflow of patient BP self-measurement in a primary care clinic. | Qualitative and quantitative | 82% of the patients were satisfied with using the kiosk for self-management of their blood pressure. Although they were initially worried about the accuracy of the existing technology, but after some time, they gained confidence in the accuracy of the technology. Also, patients and providers reported many benefits, including the following: ease of use, improved communication between the patient and the provider, and saving time. |
| 15 | ([Alnosayan et al., 2017](#_ENREF_4)) | US | This study aimed to understand challenges and provide design considerations for a personalized mHealth system that could effectively support heart failure (HF) patients after they transition into the home environment. | Qualitative and quantitative | Motivational factors: increasing patient adherence, recall, ease of access, increasing patient awareness, improving patient-provider communication, ability to identify individual patterns, reducing costs, helping the treatment process, and helping providers interpret information.  Inhibiting factors: reliability, lack of technical support, and lack of immediate problem-solving. |
| 16 | )[Dou et al., 2017](#_ENREF_11)) | China | The aim of this study was to develop and test a theoretical model to predict and explain the factors influencing patients’ acceptance of smartphone health technology for chronic disease management. | quantitative | **Motivational** **factors**: perceived usefulness, ease of use, necessary technical support, self-efficacy, and encouraging the physician to use technologies.  **Inhibiting** **factors**: resistance to change, perceived health threat, interest in face-to-face communication, previous experience using technology, lack of trust. |
| 17 | ([Peters et al., 2017](#_ENREF_37)) | 0000000 | The aim of this study was to design and implement a new phone call- and short message service text messaging-based intervention, Epharmix’s EpxHypertension, in a quality improvement project that demonstrates the feasibility of this system for BP control in a family medicine setting. | Feasibility study | **Motivating** **factors**: improving blood pressure control, regular monitoring and better treatment follow-up, better patient-provider communication, user-friendliness, ease of access, increasing access to treatment, reducing in-person visits, saving time and money, providing information and voice reminders.  **Inhibiting** **factors**: the high cost of providing technologies, spending time for installation and training of users, old age, low health literacy, low accuracy of technologies, lack of individual ability of the patient. |
| 18 | ([Rhoads et al., 2017](#_ENREF_39)) | US | Why some women with preeclampsia chose remote patient monitoring in the postpartum period and why some women refused to participate in it, as well as assessing the feasibility of remote patient monitoring and the infrastructure required for it. | Qualitative and quantitative | **Motivating** **factors**: organizational support, sufficient access to resources, having sufficient knowledge, family support, helping to better control treatment, helping with quick diagnosis, ease of use, effective training to work with the system, comprehensibility, comfortable and easy interaction with the system, flexible Being a system, the skill of using technology, a positive attitude towards the system, reducing the complications of the disease, helping to follow the treatment, increasing the feeling of relaxation and reducing worries, supporting providers, saving costs, increasing the communication of the care team, reducing the risk of patient non-adherence to treatment. , increasing patient satisfaction  **Inhibiting** **factors**: fear of technology, lack of time, lack of privacy, disruption of daily activities, lack of motivation, increased patient anxiety, geographical location (patients living in villages), lack of necessary infrastructure |
| 19 | ([Haynes & Kim, 2017](#_ENREF_21)) | US | 1) Assessing the strengths and weaknesses of the OnPoint application, a mobile phone system for managing complex chronic diseases 2) Identifying the functions and characteristics of application acceptance by patients and care team members. | Qualitative | **Motivating** **factors**: providing up-to-date information, being technologically literate, remembering and providing reminders from technology, helping with data organization, easy sharing of information, promoting awareness and knowledge, better tracking treatment, integrating existing technologies together, and integrating them. Strong technology management, user-centered, collaborative, and flexible design, and system optimization with different types of users.  **Inhibiting** **factors**: old age, low computer skills, disease severity, privacy concerns. |
| 20 | ([E. C. Morrissey et al., 2018](#_ENREF_30)) | Ierland | To explore GPs’ perspectives of self-management technology to support medication adherence and blood pressure control in patients with hypertension. | Qualitative | **Motivating** **factors**: availability of services, computer skills, level of familiarity with technologies, motivation, access to up-to-date and correct information, correct management of telehealth technologies, government support, and policy making.  **Inhibiting** **factors**: creating anxiety, lack of trust, low self-efficacy, lack of sufficient training, increasing medical and legal responsibility, increasing workload, performing activities outside working hours, and lack of resources. |
| 21 | )[Morton et al., 2018](#_ENREF_31)) | UK | This qualitative study aimed to explore perceived burdens and benefits for patients using a digital self-management intervention for reducing high blood pressure. A secondary aim was to further our understanding of how best to capture burdens and benefits when evaluating health interventions. | Qualitative/RCT | **Motivating** **factors**: convenience and ease of use, increased patient confidence, better disease control, lifestyle change, increased drug adherence, better side effect management, increased patient awareness, provide support, cost and time saving, self-efficacy, and availability.  **Inhibiting** **factors**: lack of motivation, lack of self-monitoring confidence, lack of sufficient knowledge, increased patient stress, and resistance to change. |
| 22 | )[Eimear C Morrissey et al., 2018](#_ENREF_29)) | Ierland | The aim of this study was to explore patients’ perspectives on smartphone apps to improve medication adherence in hypertension. | Qualitative | **Inhibiting** **factors**: low computer skills, lack of attention to users' needs in system design, lack of interest, lack of sufficient training, lack of knowledge and awareness of technologies, increased patient stress, lack of confidence in self-monitoring, age, privacy, data security, government support and appropriate policing, providing frequent nagging reminders.  **Motivational** **factors**: appropriate output (providing a blood pressure chart), having motivation, having previous experience of using it, believing in individual abilities, creating a sense of power, supporting providers, and encouraging the doctor to use technology. |
| 23 | ([Albrecht et al., 2018](#_ENREF_1)) | Canada | The objective of this qualitative study was to examine the usability and acceptability of a home BP telemonitoring device among senior citizens. | Qualitative | **Inhibiting** **factors**: lack of confidence in self-monitoring, lack of proper design, lack of necessary technical skills, old age, low knowledge and awareness, and limited technology literacy.  **Motivating** **factors**: family support, user-friendliness, appropriate design of the system output, ease of use, acceptability, and usefulness of the system, and provision of voice reminders. |
| 24 | ([Portz et al., 2019](#_ENREF_38)) | US | The aim of this study was to use the Technology Acceptance Model (TAM) as a framework for qualitatively describing the UI and UX, intent to use, and use behaviors among older patients with MCC. | Qualitative descriptive | **Motivating** **factors**: ease of use, perceived usefulness, improved provider-patient communication, saving time and money, and receiving up-to-date information.  **Inhibiting** **factors**: inappropriate user interface design, fear of technology, low self-efficacy, low computer skills, old age, incomprehensible technology, creating confusion, lack of proper technical support, the severity of illness, desire to meet in person, previous negative user experience, data security, Lack of interest, lack of knowledge and awareness. |
| 25 | ([Nelissen et al., 2018](#_ENREF_33)) | Nigeria | Feasibility of pharmacy-based blood pressure care using mHealth in Lagos | Mix-methode | **Motivating** **factors**: increasing access, increasing patient adherence, helping the treatment process, regular follow-up of the disease, providing financial incentives to providers, supporting providers, and recommending providers to use technologies.  **Inhibiting** **factors**: lack of integration of technologies with the work process, not user-friendly, desire for face-to-face referrals, reimbursement problems, implementation cost. |
| 26 | )[Chérrez-Ojeda et al., 2019](#_ENREF_8)) | Ecuador | This study was designed to assess the use and preferences for information and communication technologies (ICTs) among patients with hypertension in Ecuador. | Cross-sectional | **Motivating** **factors**: facilitating the exchange and sharing of information, increasing problem-solving skills, increasing self-confidence, saving money and time, appropriate technical support, providing equitable health services to all regions, appropriate design, the ability to personalize, and improving individual habits. Get information quickly.  **Inhibiting** **factors**: Internet problem, low education, old age, low interest, geographical areas (where one lives), economic conditions. |
| 27 | )[Xydopoulos et al., 2019](#_ENREF_50)) | UK | The aim of this study was to undertake a health economic analysis of HBPM compared with traditional monitoring in hypertensive pregnant women. | RCT | The results show that compared to the traditional monitoring path in hypertensive pregnancies, without endangering the health of the mother, the fetus, or the baby, the number of outpatient visits of the patient is reduced and costs are saved. |
| 28 | )[Xiao et al., 2019](#_ENREF_49)) | Chine | This was a feasibility study aimed to understand patient and medical practitioners’ acceptance and experience of a mobile-phone based platform for the management of hypertensive patients. | Mix-methode | **Motivating** **factors**: ease of use, reminders, design according to users' needs, user-friendliness, helping the treatment process, increasing knowledge and awareness, lack of time and place restrictions, helping providers in the decision-making process, improving service quality, up-to-date information and accurate. |
| 29 | )[Grant et al., 2019](#_ENREF_17)) | UK | Evaluation of facilitators and barriers to self- and telemonitoring interventions for hypertension within the Telemonitoring and Self-monitoring in Hypertension (TASMINH4) trial. | Qualitative | **Motivating** **factors**: simplicity, ease of use, quick access, increasing patient dynamism, self-efficacy, increasing independence and autonomy, increasing patient participation, improving communication between doctor and patient, saving time and money, helping in decision making, and quick diagnosis.  **Inhibiting** **factors**: reluctance to use technologies, lack of confidence in self-monitoring, fear of technology, old age, low self-confidence, data security, privacy, and lack of confidence in the accuracy of received information. |
| 30 | ([Dugelay et al., 2019](#_ENREF_12)) | France | To explore the perception of home blood pressure monitoring (HBPM) by general practitioners (GPs) in everyday practice in order to identify facilitators and barriers to its implementation in daily practice. | Qualitative | **Inhibiting** **factors**: lack of confidence in the presented materials, increase in workload, increase in patient anxiety, lack of resources, cost of providing technologies, lack of time and human resources, lack of sufficient training, lack of medical knowledge, increase in medical responsibility, increase in legal responsibility, creating false sense Self-management in patients and decision-making without consulting a doctor.  **Motivational** **factors**: increasing the understanding and awareness of patients, improving the treatment process. |
| 31 | ([Allen et al., 2019](#_ENREF_3)) | US | This study explores the mechanisms by which MyBP, an automated SMS-facilitated home blood pressure monitoring (HBPM) program, helps facilitate healthy behavior changes. | Qualitative | **Motivational** **factors**: increasing knowledge about blood pressure, providing reminders, improving personal habits, and increasing self-confidence.  **Inhibiting** **factors**: creating anxiety, lack of self-monitoring confidence. |
| 32 | ([Hoppe et al., 2019](#_ENREF_22)) | US | Investigate feasibility of telehealth with remote blood pressure monitoring for management of hypertension in postpartum women at risk of severe hypertension after hospital discharge. | Prospective cohort usability study | **Inhibiting** **factors**: Data security, privacy, disease severity, and time-consuming. |
| 33 | ([Barsky et al., 2019](#_ENREF_5)) | Canada | The study aim was to assess the effect on the difference in blood pressure reduction of active hypertension management messages or passive health behavior messages. The system was designed to be implemented in remote areas with wireless availability. | Mix-methode | **Motivating** **factors**: social support, family support, stress reduction, provision of up-to-date information, user-friendliness, improvement of personal habits, treatment follow-up, improvement of communication, increase in self-confidence, help in self-efficacy, more patient cooperation in treatment, increase in awareness, useful For hard-to-reach areas.  **Inhibiting** **factors**: technical problems of the system, privacy. |
| 34 | ([Islam et al., 2019](#_ENREF_23)) | Australia | This study aimed to validate a wrist-worn cuffless wearable BP device (Model T2; TMART Technologies Limited) and assess its acceptability among users and health care professionals. | Mix-methode | **Motivating** **factors**: simplicity, proper design, high accuracy, validation of technologies, the possibility of continuous monitoring at any time and place, better effectiveness of treatment, increased adherence, and ability to share data.  **Inhibiting** **factors**: the high cost of providing technology, privacy, data security, and lack of confidence in the provided content. |
| 35 | )[Silveira et al., 2019](#_ENREF_43)) | Berzeli | The goal of the research was to evaluate the feasibility, usability, and utility of a CDSS, TeleHAS (tele–hipertensão arterial sistêmica, or arterial hypertension system), in the care of patients with hypertension in the context of a primary care setting in a middle-income country. | Mix-methode | **Motivational** **factors**: improving blood pressure control, providing up-to-date information, having motivation and interest, simplicity and ability to understand the system, providing warnings and reminders, younger workforce, high technical skills and knowledge, ease of access, ease of use, sufficient training, ability to use Offline from the system, user-friendly, suitable screen design.  **Inhibiting** **factors**: inappropriate design, increase in workload, lack of time for data entry, increase in patients' stress, technical problems, lack of proper technical support, internet speed problems, resistance to change, high cost of technology, lack of integration of existing technologies. Feeling confused when working with the system, fear the possibility of patient safety. |
| 36 | ([Nichols et al., 2019](#_ENREF_34)) | 00000 | Effectiveness of a mobile health technology intervention for the management of hypertension among stroke survivors | Mix-methode | **Motivating** **factors**: quick access, increasing self-efficacy, continuous disease monitoring, family support, reducing patient anxiety, changing eating habits, providing reminders and warnings, increasing patient awareness, saving time and money, receiving accurate and up-to-date information, and increasing patient adherence. To treatment, social support, comprehensibility, ease of technology, provision of training, provision of necessary technical support, availability of passwords, increased medication adherence, and better disease control.  **Inhibiting** **factors**: patient confusion, physical limitations of the patient, lack of technology literacy, lack of interest, geographical location, and high cost of implementation. |
| 37 | ([Steinman et al., 2020](#_ENREF_45)) | Cambogia | This study aimed to understand the facilitators and barriers to chronic disease management and the acceptability, appropriateness, and feasibility of mHealth to support chronic disease management and strengthen community-clinical linkages to existing services. | Qualitative | **Motivational** **factors**: saving time, affordability, ease of access, providing reminders, social and family support, government support, increasing self-efficacy, and belief in individual abilities.  **Inhibiting** **factors**: low knowledge and awareness (literacy), low computer skills, old age, physical problems, lack of resources, high expectations of users from technology, financial problems, limited insight into the disease, and more trust in face-to-face referrals. |
| 38 | ([Cairns et al., 2020](#_ENREF_7)) | UK | The process of implementation was explored during a pilot randomised controlled trial evaluating postpartum blood pressure self-management in women with medicated hypertensive disorders of pregnancy. | Interview | **Motivating** **factors**: better control of blood pressure, increasing responsibility, ease of access, absence of time and place limitations, help in the continuous follow-up of treatment, improvement of the quality of the treatment process, improvement of patient-provider communication, support of providers and advisors to use technologies, support Social, family support, providing alerts and reminders.  **Inhibiting** **factors**: the feeling of control, trust, limited insight into the disease, increased stress of the patient. |
| 39 | ([Jongsma et al., 2020](#_ENREF_25)) | Netherland | The objectives were twofold: (1) to explore the experiences of Dutch women who had an increased risk of HDP with a blended care approach (mHealth combined with face-to-face care) for remote self-monitoring of blood pressure and preeclampsia symptoms and (2) to formulate recommendations for the use and integration of mHealth in clinical care. | Qualitative and quantitative | **Motivating** **factors**: ease of use, reduction in the number of visits, prevention of disease complications, help in early diagnosis, design suitable to the user's needs, perceived usefulness, user-friendliness, providing reminders and warnings, increasing autonomy and responsibility, increasing awareness, supporting providers. , improving patient-provider communication, helping to monitor and follow up on treatment, and experience of use.  **Inhibiting** **factors**: security, lack of confidence in the presented materials, limited technology literacy, anxiety, lack of technical support, willingness to meet in person, the severity of the disease, lack of necessary guides to using the system, lack of appropriate policies, privacy, ethical challenges, age, Level of Education. |
| 40 | ([van den Heuvel et al., 2020](#_ENREF_47)) | Netherland | This study aims to assess the current practice and attitudes concerning home-based monitoring (with daily home visits by professionals) and telemonitoring (using devices and the internet for daily self-recorded measurements) in high-risk pregnancies requiring maternal and fetal monitoring in the Netherlands. | Quantitative | **Motivating** **factors**: not jeopardizing patient safety, user satisfaction, affordability, daily and continuous monitoring of the patient, saving time, availability, reducing care costs, reducing emotional burden, reducing stress, and increasing patient self-efficacy.  **Inhibiting** **factors**: willingness to visit in person, resistance to change, financial problems, problems related to staff capacity, inability to follow instructions by the patient, inability to understand the system, lack of family support, lack of social support, the possibility of delay in providing help, problems Technical system, refund problems. |
| 41 | )[Jahan et al., 2020](#_ENREF_24)) | Bangladesh | The study aimed to develop awareness and knowledge in order to enhance lifestyle behavior changes among individuals with HTN in a rural community of Bangladesh by using health education and mobile health (mHealth) technology (SMS text messaging). | RCT | **Motivational** **factors**: changing personal habits, lowering blood pressure, increasing quality of life, increasing awareness.  **Inhibiting** **factors**: lack of ease of access, inability to use easily, lack of quick feedback of technologies, lack of access to technologies, limited resources, high cost of technologies, poverty. |
| 42 | )[Schoenthaler et al., 2020](#_ENREF_41)) | US | This study aims to develop and evaluate the acceptability and preliminary efficacy of a tailored mHealth adherence intervention versus attention control (AC) on medication adherence, systolic blood pressure (SBP), diastolic blood pressure (DBP), and hemoglobin A1c (HbA1c) at 3 months in 42 Black patients with uncontrolled HTN and/or T2D who were initially nonadherent to their medications. | Mix-methode | **Motivating** **factors**: provider support, patient-provider communication, providing effective information, changing individual habits, changing lifestyle, and ease to use.  **Inhibiting** **factors**: too much busyness, lack of interest, disruption of the daily schedule, concern about adverse effects, desire for face-to-face consultations, forgetting to use technology, lack of clinical knowledge, lack of confidence in the presented materials, wrong attitude, lack of personal motivation. |
| 43 | )[Treskes et al., 2020](#_ENREF_46)) | Netherland | Does the use of smartphone technology have an effect on improved blood pressure regulation in patients after myocardial infarction and is it feasible to implement? | RCT | **Inhibiting** **factors**: increased fear and anxiety due to high exposure to the disease, fear of technology, desire to follow up in person, and lack of motivation. |
| 44 | )[Song et al., 2021](#_ENREF_44)) | Chine | This study aims to explore patients’ perceptions of mHealth services and the mechanisms by which the services support them to self-manage their hypertension. | Qualitative | **Motivating** **factors**: quick and easy access to services, support of providers, confirmation and confirmation of content information by the provider, increasing self-efficacy and self-management of patients, providing reminders, creating a competitive environment between organizations, lack of time and place restrictions, cost-effectiveness, Helping to improve treatment processes, helping to evaluate treatment processes, continuous evaluation, and measurement of information sources, increasing patient dynamism and responsibility, increasing knowledge and awareness.  **Inhibiting** **factors**: lack of trust in the presented materials, low accuracy of technologies. |
| 45 | )[Walcott-Bryant et al., 2021](#_ENREF_48)) | Kneya | The aim of this study is to assess stakeholders’ perspectives on the challenges associated with the management of hypertension in the Kenyan private health care sector and to derive recommendations for the design and functionality of a digital health solution for addressing the care continuity and quality challenges in the management of hypertension. | Qualitative | **Motivating** **factors**: cost-effectiveness, training and introduction of technologies before introduction, better follow-up of treatment, increasing patient adherence to treatment, improving knowledge, providing financial incentives, improving infrastructure, and creating rules and regulations. |
| 46 | )[Alessa et al., 2020](#_ENREF_2)) | Saudi Arabia | This study aims to evaluate the acceptance and usability of the selected app in the Saudi context. | Qualitative and quantitative | **Motivating** **factors**: appropriate user interface, ease of use, design tailored to the user's needs, improving the patient's understanding of his health status, helping to manage the disease, providing up-to-date and useful information, designing a simple and intuitive system output (presenting a diagram), increasing adherence to Treatment, providing reminders.  **Inhibiting** **factors**: lack of technical support, old age, low education, lack of previous user experience, internet problems, stress. |
| 47 | )[Park et al., 2021](#_ENREF_35)) | US | The aims of this study are to characterize the adherence of Medicaid patients with hypertension to daily telemonitoring, identify the impacts of adherence reminder calls, and investigate associations with blood pressure control. | RCT | Remote monitoring is effective in reducing blood pressure and patients achieve high levels of adherence to treatment with the help of remote monitoring, according to the results of the study, providing reminders is also effective in increasing patients' adherence. |
| 48 | )[Bozorgi et al., 2021](#_ENREF_6)) | Iran | In view of the increased use of mobile health in medicine, the present study evaluated the effect of a self-management application on patient adherence to hypertension treatment. | RCT | **Motivational** **factors**: increasing adherence to treatment, increasing knowledge and awareness of patients, increasing self-efficacy, providing medication reminders, increasing treatment follow-up, optimal blood pressure control, improving personal habits, providing sufficient information, personal interest, ease of use, support from providers, family support. |
| 49 | )[Nau et al., 2021](#_ENREF_32)) | Australia | This pilot study aimed to evaluate the acceptability and feasibility of integrating a mobile health intervention into primary care to support patients with improving lifestyle behaviours for high blood pressure. | Mix-methode | **Motivating** **factors**: easy use, changing lifestyle, user-friendliness of the system, saving messages and information, providing reminders, motivation, and interest, receiving messages on time, providing more up-to-date and more accurate and detailed information, facilitating information sharing, insight deep, personalization ability, awareness raising, proper design. |
| 50 | )[Citoni et al., 2022](#_ENREF_10)) | Italy | Strengths and limitations of telemedicine in the clinical management of hypertension during the COVID-19 pandemic | Qualitative | **Motivating** **factors**: ease of information exchange, help to prevent and better treatment of the disease, help to continuously follow up the disease, help to train the providers, help to research, improve the communication between the provider and the patient, especially in quarantine conditions, improve the awareness of patients, prevent medical errors. , helping self-management, increasing self-efficacy, saving time and money, increasing participation, increasing patient adherence, changing lifestyles, availability, and attitude, and recommending providers to use these technologies.  **Inhibiting** **factors**: lack of validation of technologies, lack of sufficient training in the use of technologies, time-consuming, lack of integration with the clinical work process, increased anxiety and worry of patients, and old age. |

**Appendix 2: Identified factors (Motivational factors)**

Table 2. Motivational factors

| **References** | **Motivational factors** | **Groups** |
| --- | --- | --- |
| [[1-19](#_ENREF_1)] | Improving patient-provider communication and increasing patient participation. | Organizational |
| [[1](#_ENREF_1), [3](#_ENREF_3), [4](#_ENREF_4), [9](#_ENREF_9), [11-13](#_ENREF_11), [16](#_ENREF_16), [20-24](#_ENREF_20)] | Social and family support. |  |
| [[11](#_ENREF_11), [19](#_ENREF_19), [23](#_ENREF_23), [25](#_ENREF_25), [26](#_ENREF_26)] | Government support. |  |
| [[27](#_ENREF_27)] | Organizational support. |  |
| [[19](#_ENREF_19)] | High work commitment. |  |
| [[4](#_ENREF_4), [6](#_ENREF_6), [14](#_ENREF_14), [15](#_ENREF_15), [18](#_ENREF_18), [20](#_ENREF_20), [26-33](#_ENREF_26)] | Cooperation and support of providers and advice on the use of technologies. |  |
| [[19](#_ENREF_19), [33](#_ENREF_33)] | Creating a competitive environment between organizations. |  |
| [[19](#_ENREF_19), [31](#_ENREF_31), [34](#_ENREF_34)] | Providing financial incentives. |  |
| [[19](#_ENREF_19), [34](#_ENREF_34)] | Improving cooperation and coordination between providers. |  |
| [[2](#_ENREF_2), [4](#_ENREF_4), [13](#_ENREF_13), [27](#_ENREF_27), [32](#_ENREF_32), [33](#_ENREF_33), [35-39](#_ENREF_35)] | Helping to improve treatment processes and quality of care. |  |
| [[33](#_ENREF_33)] | Helping to evaluate and compare treatment processes. |  |
| [[3](#_ENREF_3), [4](#_ENREF_4), [6](#_ENREF_6), [7](#_ENREF_7), [14](#_ENREF_14), [16](#_ENREF_16), [21](#_ENREF_21), [24](#_ENREF_24), [28](#_ENREF_28), [31](#_ENREF_31), [34](#_ENREF_34), [37](#_ENREF_37), [38](#_ENREF_38)] | Regular monitoring and better treatment follow-up. |  |
| [[2](#_ENREF_2)] | Ability to identify individual patterns. |  |
| [[2](#_ENREF_2), [10](#_ENREF_10), [27](#_ENREF_27), [39](#_ENREF_39), [40](#_ENREF_40)] | Assisting providers in interpreting information and making decisions and early diagnosis |  |
| [[2](#_ENREF_2), [13](#_ENREF_13), [14](#_ENREF_14), [16](#_ENREF_16), [19](#_ENREF_19), [41](#_ENREF_41), [42](#_ENREF_42)] | Reducing patient readmissions and in-person referrals |  |
| [[25](#_ENREF_25)] | Proper management of telehealth technologies |  |
| [[6](#_ENREF_6), [14](#_ENREF_14), [16](#_ENREF_16), [31](#_ENREF_31), [43](#_ENREF_43)] | Prevention and reduction of disease complications |  |
| [[19](#_ENREF_19)] | The degree of preparation of organization members |  |
| [[19](#_ENREF_19)] | Organization's readiness for change |  |
| [[19](#_ENREF_19)] | Ability to work remotely |  |
| [[35](#_ENREF_35)] | The possibility of providing fair health services to all places |  |
| [[4](#_ENREF_4), [16](#_ENREF_16), [22](#_ENREF_22), [26](#_ENREF_26), [28](#_ENREF_28), [38](#_ENREF_38), [40](#_ENREF_40), [41](#_ENREF_41), [44](#_ENREF_44)] | Improving control and management of blood pressure |  |
| [[6](#_ENREF_6), [11](#_ENREF_11), [13](#_ENREF_13), [37](#_ENREF_37)] | Integration and integration of existing technologies |  |
| [[6](#_ENREF_6)] | Preventing medical errors |  |
| [[3](#_ENREF_3)] | Being useful for hard-to-reach are |  |
| [[6](#_ENREF_6)] | Research assistanc |  |
| [[19](#_ENREF_19)] | Increasing job satisfactio |  |
| [[6](#_ENREF_6)] | Young workforce |  |
| [[4](#_ENREF_4), [6](#_ENREF_6), [9](#_ENREF_9), [11](#_ENREF_11), [14](#_ENREF_14), [15](#_ENREF_15), [18-20](#_ENREF_18), [22](#_ENREF_22), [27-29](#_ENREF_27), [31](#_ENREF_31), [32](#_ENREF_32), [35-37](#_ENREF_35), [39](#_ENREF_39), [40](#_ENREF_40), [42](#_ENREF_42), [44-46](#_ENREF_44)] | Saving costs | Economic |
| [[20](#_ENREF_20)] | Providing financial resources |  |
| [[9](#_ENREF_9), [46](#_ENREF_46)] | Providing free services |  |
| [[20](#_ENREF_20)] | Benefit cost |  |
| [[46](#_ENREF_46)] | Annual income |  |
| [[2](#_ENREF_2), [4](#_ENREF_4), [11](#_ENREF_11), [12](#_ENREF_12), [19](#_ENREF_19), [21](#_ENREF_21), [22](#_ENREF_22), [25](#_ENREF_25), [28](#_ENREF_28), [33](#_ENREF_33), [37](#_ENREF_37), [39](#_ENREF_39), [43](#_ENREF_43), [44](#_ENREF_44), [47](#_ENREF_47), [48](#_ENREF_48)] | Remembering and providing audio reminders by technologies | Technical |
| [[1](#_ENREF_1), [7](#_ENREF_7), [11](#_ENREF_11), [13](#_ENREF_13), [14](#_ENREF_14), [19](#_ENREF_19), [30](#_ENREF_30), [35](#_ENREF_35), [40](#_ENREF_40), [42](#_ENREF_42), [44](#_ENREF_44), [46](#_ENREF_46), [47](#_ENREF_47)] | Appropriate and user-oriented design |  |
| [[19](#_ENREF_19), [27](#_ENREF_27), [37](#_ENREF_37)] | Flexible design |  |
| [[3](#_ENREF_3), [7](#_ENREF_7), [14](#_ENREF_14), [16](#_ENREF_16), [20](#_ENREF_20), [31](#_ENREF_31), [39](#_ENREF_39), [44](#_ENREF_44), [46](#_ENREF_46), [47](#_ENREF_47)] | User-friendliness |  |
| [[6](#_ENREF_6), [11](#_ENREF_11), [12](#_ENREF_12), [21](#_ENREF_21), [30](#_ENREF_30), [36](#_ENREF_36)] | Training and introduction of technologies before introduction |  |
| [[3](#_ENREF_3), [7](#_ENREF_7), [15](#_ENREF_15), [17](#_ENREF_17), [18](#_ENREF_18), [21](#_ENREF_21), [25](#_ENREF_25), [28](#_ENREF_28), [37](#_ENREF_37), [40](#_ENREF_40), [44](#_ENREF_44), [46](#_ENREF_46), [47](#_ENREF_47)] | Providing up-to-date, correct and accurate information |  |
| [[21](#_ENREF_21)] | Technical support of technologies |  |
| [[9](#_ENREF_9)] | Availability of technologies |  |
| [[19](#_ENREF_19), [37](#_ENREF_37)] | Cooperation between user and system developer |  |
| [[37](#_ENREF_37)] | Continuous assessment and measurement of information sources |  |
| [[37](#_ENREF_37)] | Help to organize data |  |
| [[6](#_ENREF_6), [13](#_ENREF_13), [32](#_ENREF_32), [34](#_ENREF_34), [35](#_ENREF_35), [37](#_ENREF_37), [44](#_ENREF_44), [47](#_ENREF_47)] | Facilitating the exchange and sharing of information and experiences |  |
| [[37](#_ENREF_37), [44](#_ENREF_44)] | Integration of existing technologies and their integration |  |
| [[37](#_ENREF_37)] | Optimizing the system with different types of users |  |
| [[34](#_ENREF_34)] | Access to patient history and information |  |
| [[5](#_ENREF_5), [9](#_ENREF_9), [16](#_ENREF_16), [32](#_ENREF_32), [33](#_ENREF_33), [45](#_ENREF_45)] | Increasing the accuracy of technologies |  |
| [[1](#_ENREF_1), [5](#_ENREF_5), [7](#_ENREF_7), [10-12](#_ENREF_10), [14](#_ENREF_14), [16-18](#_ENREF_16), [21](#_ENREF_21), [27](#_ENREF_27), [29](#_ENREF_29), [38-40](#_ENREF_38), [45](#_ENREF_45), [49](#_ENREF_49)] | Simplicity and easy interaction and ease of use of the system |  |
| [[7](#_ENREF_7), [47](#_ENREF_47)] | Simplifying and personalizing technologies |  |
| [[13](#_ENREF_13)] | Instant and fast reading of information |  |
| [[11](#_ENREF_11), [15](#_ENREF_15)] | The possibility of making an appointment |  |
| [[6](#_ENREF_6), [12](#_ENREF_12), [44](#_ENREF_44)] | Validation of technologies |  |
| [[9](#_ENREF_9), [10](#_ENREF_10), [14](#_ENREF_14), [17](#_ENREF_17), [30](#_ENREF_30), [38](#_ENREF_38), [41](#_ENREF_41)] | Increasing data security |  |
| [[1](#_ENREF_1), [11](#_ENREF_11), [30](#_ENREF_30), [40](#_ENREF_40)] | Proper design of system output |  |
| [[1](#_ENREF_1)] | Acceptability and usefulness of the system |  |
| [[16](#_ENREF_16), [40](#_ENREF_40), [44](#_ENREF_44)] | Appropriate user interface design |  |
| [[11](#_ENREF_11)] | Presentation of the daily program |  |
| [[37](#_ENREF_37)] | Optimizing the system with different types of users |  |
| [[25](#_ENREF_25), [37](#_ENREF_37)] | Correct management of technologies |  |
| [[2](#_ENREF_2), [13](#_ENREF_13)] | System and information reliability |  |
| [[8](#_ENREF_8), [21](#_ENREF_21), [27](#_ENREF_27), [44](#_ENREF_44)] | Understandability |  |
| [[31](#_ENREF_31)] | Increasing patient confidence in technology |  |
| [[44](#_ENREF_44)] | The ability to use the system offline |  |
| [[21](#_ENREF_21)] | Existence of password |  |
| [[2](#_ENREF_2), [4](#_ENREF_4), [6](#_ENREF_6), [10](#_ENREF_10), [11](#_ENREF_11), [15](#_ENREF_15), [19](#_ENREF_19), [21](#_ENREF_21), [24-26](#_ENREF_24), [31](#_ENREF_31), [33](#_ENREF_33), [35](#_ENREF_35), [38](#_ENREF_38), [44](#_ENREF_44), [46](#_ENREF_46), [47](#_ENREF_47)] | Quick and easy access to information and services |  |
| [[1](#_ENREF_1), [3](#_ENREF_3), [7](#_ENREF_7), [11](#_ENREF_11), [21](#_ENREF_21), [26](#_ENREF_26), [29](#_ENREF_29), [31](#_ENREF_31), [39](#_ENREF_39), [44-47](#_ENREF_44)] | Saving time |  |
| [[2](#_ENREF_2), [6](#_ENREF_6), [11](#_ENREF_11), [20-22](#_ENREF_20), [25-28](#_ENREF_25), [31](#_ENREF_31), [34](#_ENREF_34), [38](#_ENREF_38), [40](#_ENREF_40), [48](#_ENREF_48)] | Increasing patient adherence to treatment and medication |  |
| [[3](#_ENREF_3), [6](#_ENREF_6), [9-11](#_ENREF_9), [13](#_ENREF_13), [21](#_ENREF_21), [23](#_ENREF_23), [24](#_ENREF_24), [26](#_ENREF_26), [28-31](#_ENREF_28), [33](#_ENREF_33), [46](#_ENREF_46)] | Increasing self-efficacy and belief in personal abilities |  |
| [[3](#_ENREF_3), [6](#_ENREF_6), [7](#_ENREF_7), [9](#_ENREF_9), [11](#_ENREF_11), [15](#_ENREF_15), [19-21](#_ENREF_19), [26](#_ENREF_26), [28](#_ENREF_28), [34](#_ENREF_34), [36](#_ENREF_36), [39](#_ENREF_39), [47](#_ENREF_47), [49](#_ENREF_49)] | Improving knowledge and awareness |  |
| [[3](#_ENREF_3), [6](#_ENREF_6), [11](#_ENREF_11), [18](#_ENREF_18), [21](#_ENREF_21), [31](#_ENREF_31), [35](#_ENREF_35), [43](#_ENREF_43), [47](#_ENREF_47), [49](#_ENREF_49)] | Helping to improve personal and eating habits (lifestyle change) |  |
| [[20](#_ENREF_20), [21](#_ENREF_21), [25](#_ENREF_25), [27](#_ENREF_27), [37](#_ENREF_37), [46](#_ENREF_46)] | Familiarity with technology |  |
| ([[4](#_ENREF_4), [7](#_ENREF_7), [9](#_ENREF_9), [10](#_ENREF_10), [14](#_ENREF_14), [15](#_ENREF_15), [33](#_ENREF_33)] | Increasing patient dynamism and responsibility |  |
| [[11](#_ENREF_11), [14](#_ENREF_14), [17](#_ENREF_17), [26](#_ENREF_26), [29](#_ENREF_29), [30](#_ENREF_30)] | The experience of the patient's previous use of technologies |  |
| [[4](#_ENREF_4), [33](#_ENREF_33), [39](#_ENREF_39)] | Absence of time and place limitations |  |
| ([[20](#_ENREF_20), [33](#_ENREF_33), [43](#_ENREF_43)] | Increasing self-management |  |
| [[33](#_ENREF_33)]  ([[3](#_ENREF_3), [9](#_ENREF_9), [21](#_ENREF_21), [24](#_ENREF_24), [27](#_ENREF_27)] | Confirmation and verification of information by the provider |  |
|  | Reducing emotional load and stress |  |
| [[4](#_ENREF_4), [11](#_ENREF_11), [17](#_ENREF_17), [26](#_ENREF_26), [29](#_ENREF_29)] | Perceived usefulness |  |
| [[3](#_ENREF_3), [35](#_ENREF_35), [43](#_ENREF_43)] | Increasing self-confidence |  |
| [[35](#_ENREF_35)] | Increasing problem solving skills |  |
| [[7](#_ENREF_7), [9](#_ENREF_9), [11](#_ENREF_11), [25](#_ENREF_25), [30](#_ENREF_30), [44](#_ENREF_44)] | Sufficient motivation |  |
| ([[6](#_ENREF_6), [15](#_ENREF_15), [20](#_ENREF_20), [27](#_ENREF_27), [50](#_ENREF_50)] | Positive attitude |  |
| [[40](#_ENREF_40)] | Improving the patient's understanding of his health status |  |
| [[10](#_ENREF_10), [14](#_ENREF_14)] | Increasing independence and autonomy |  |
| [[24](#_ENREF_24), [27](#_ENREF_27)] | Increasing patient satisfaction |  |
| [[30](#_ENREF_30)] | Creating a sense of power |  |
| [[20](#_ENREF_20)] | Increasing the quality of life |  |
| [[7](#_ENREF_7)] | Helping to keep personal identity and type of disease confidential | Legal/Moral |

**Appendix 3: Identified factors (Motivational factors)**

Table 3. Inhibitory factors

| **References** | **Inhibitory factors** | **Groups** |
| --- | --- | --- |
| [[13](#_ENREF_13), [19](#_ENREF_19), [25](#_ENREF_25), [32](#_ENREF_32), [36](#_ENREF_36), [39](#_ENREF_39), [44](#_ENREF_44)] | Increasing workload | Organizational |
| [[24](#_ENREF_24), [26](#_ENREF_26), [29](#_ENREF_29), [44](#_ENREF_44)] | Resistance to change |  |
| [[19](#_ENREF_19), [23](#_ENREF_23), [25](#_ENREF_25), [29](#_ENREF_29), [49](#_ENREF_49)] | lack of resources |  |
| [[14](#_ENREF_14), [15](#_ENREF_15), [19](#_ENREF_19), [30](#_ENREF_30), [32](#_ENREF_32), [34](#_ENREF_34)] | Lack of appropriate policies |  |
| ([[49](#_ENREF_49)] | Delay in providing assistance to the patient |  |
| ([[8](#_ENREF_8), [27](#_ENREF_27)] | Lack of access to resources |  |
| [[19](#_ENREF_19)] | The need to redesign the work process |  |
| [[19](#_ENREF_19)] | Lack of proper infrastructure |  |
| [[6](#_ENREF_6), [13](#_ENREF_13), [31](#_ENREF_31)] | Failure to integrate technologies with the work process |  |
| [[43](#_ENREF_43)] | Manpower shortage |  |
| [[25](#_ENREF_25), [36](#_ENREF_36)] | Increasing medical and legal responsibility |  |
| [[25](#_ENREF_25), [32](#_ENREF_32)] | Forced to perform activities outside working hours |  |
| [[16](#_ENREF_16), [19](#_ENREF_19), [27](#_ENREF_27)] | Worrying about prolonging the installation and implementation time of the system |  |
| [[37](#_ENREF_37)] | Lack of organizational culture |  |
| [[27](#_ENREF_27), [34](#_ENREF_34)] | Lack of necessary infrastructure |  |
| [[49](#_ENREF_49)]) | Problems related to staff capacity |  |
| [[42](#_ENREF_42), [43](#_ENREF_43)] | Lack of enough time to enter data |  |
| ([[11](#_ENREF_11), [12](#_ENREF_12), [15](#_ENREF_15), [19](#_ENREF_19), [23](#_ENREF_23), [24](#_ENREF_24), [32](#_ENREF_32), [35](#_ENREF_35), [49](#_ENREF_49)] | Poverty and financial problems | Economic |
| [[16](#_ENREF_16), [21](#_ENREF_21), [29](#_ENREF_29), [31](#_ENREF_31), [32](#_ENREF_32), [38](#_ENREF_38), [44](#_ENREF_44), [49](#_ENREF_49)]  [[24](#_ENREF_24), [31](#_ENREF_31), [32](#_ENREF_32)] | The high cost of preparing and implementing technologies |  |
|  | Problems related to refunds |  |
| [[11](#_ENREF_11), [12](#_ENREF_12), [15](#_ENREF_15), [19](#_ENREF_19), [23](#_ENREF_23), [24](#_ENREF_24), [32](#_ENREF_32), [35](#_ENREF_35), [49](#_ENREF_49)] | Poverty and financial problems |  |
| [[2](#_ENREF_2), [11](#_ENREF_11), [12](#_ENREF_12), [14](#_ENREF_14), [15](#_ENREF_15), [17](#_ENREF_17), [19](#_ENREF_19), [23](#_ENREF_23), [24](#_ENREF_24), [26](#_ENREF_26), [29](#_ENREF_29), [32](#_ENREF_32), [35](#_ENREF_35), [40](#_ENREF_40), [49](#_ENREF_49)] | Lack of technical support and immediate resolution of technology problems | Technical |
| ([[17](#_ENREF_17), [21](#_ENREF_21), [44](#_ENREF_44), [46](#_ENREF_46)] | A large amount of information causes confusion |  |
| [[3](#_ENREF_3), [11](#_ENREF_11), [24](#_ENREF_24), [44](#_ENREF_44)] | Technical problems of technologies |  |
| [[11](#_ENREF_11), [35](#_ENREF_35), [40](#_ENREF_40), [44](#_ENREF_44)] | Internet problems |  |
| [[15](#_ENREF_15), [18](#_ENREF_18)] | Lack of access to technologies |  |
| [[25](#_ENREF_25)] | Providing frequent reminders that become annoying over time |  |
| [[23](#_ENREF_23)] | More expectations of users from technologies |  |
| [[49](#_ENREF_49)] | Absence of a comprehensive guide to the use of technology |  |
| [[14](#_ENREF_14)] | The incomprehensibility of technology |  |
| [[17](#_ENREF_17)] | Failure to provide sufficient information |  |
| [[15](#_ENREF_15)] | Existence of heterogeneous information |  |
| ([[8](#_ENREF_8), [11](#_ENREF_11), [12](#_ENREF_12), [17](#_ENREF_17), [18](#_ENREF_18), [20](#_ENREF_20), [25-28](#_ENREF_25), [35](#_ENREF_35), [46](#_ENREF_46), [47](#_ENREF_47), [50](#_ENREF_50)] | lack of motivation and interest | Personal |
| ([[4](#_ENREF_4), [7](#_ENREF_7), [8](#_ENREF_8), [14](#_ENREF_14), [17](#_ENREF_17), [19](#_ENREF_19), [25-27](#_ENREF_25), [35](#_ENREF_35), [40](#_ENREF_40)] | Low knowledge and awareness (education) |  |
| [[1](#_ENREF_1), [14](#_ENREF_14), [21](#_ENREF_21), [23](#_ENREF_23), [26](#_ENREF_26), [37](#_ENREF_37)] | Not having computer knowledge and skills |  |
| [[1](#_ENREF_1), [6](#_ENREF_6), [10](#_ENREF_10), [11](#_ENREF_11), [14](#_ENREF_14), [16](#_ENREF_16), [17](#_ENREF_17), [20](#_ENREF_20), [23](#_ENREF_23), [30](#_ENREF_30), [32](#_ENREF_32)] | Old age |  |
| ([[8](#_ENREF_8), [9](#_ENREF_9), [11](#_ENREF_11), [14](#_ENREF_14), [16](#_ENREF_16), [18](#_ENREF_18), [40](#_ENREF_40)] | Limited insight into the disease, and lack of health literacy |  |
| [[9-11](#_ENREF_9), [14](#_ENREF_14), [15](#_ENREF_15), [17](#_ENREF_17), [18](#_ENREF_18), [23](#_ENREF_23), [24](#_ENREF_24), [31](#_ENREF_31), [50](#_ENREF_50)] | More trust and desire for face-to-face referrals |  |
| ([[9](#_ENREF_9), [10](#_ENREF_10), [14](#_ENREF_14), [15](#_ENREF_15), [18](#_ENREF_18), [19](#_ENREF_19), [25](#_ENREF_25), [26](#_ENREF_26), [29](#_ENREF_29), [33](#_ENREF_33), [36](#_ENREF_36), [38](#_ENREF_38)] | Lack of trust in the presented materials |  |
| [[10](#_ENREF_10), [11](#_ENREF_11), [16](#_ENREF_16), [26](#_ENREF_26), [30-32](#_ENREF_30), [43](#_ENREF_43)]) | Lack of confidence in self-monitoring and personal abilities |  |
| [[6](#_ENREF_6), [13](#_ENREF_13), [14](#_ENREF_14), [25-27](#_ENREF_25), [30](#_ENREF_30), [36](#_ENREF_36), [38](#_ENREF_38), [40](#_ENREF_40)] | Increasing patient stress |  |
| [[10](#_ENREF_10), [11](#_ENREF_11), [17](#_ENREF_17), [27](#_ENREF_27), [32](#_ENREF_32), [50](#_ENREF_50)] | Fear of technology |  |
| [[14](#_ENREF_14), [15](#_ENREF_15), [17](#_ENREF_17), [37](#_ENREF_37), [43](#_ENREF_43)] | Disease severity |  |
| [[6](#_ENREF_6), [19](#_ENREF_19), [27](#_ENREF_27), [41](#_ENREF_41)] | Being time-consuming |  |
| [[21](#_ENREF_21), [23](#_ENREF_23)] | Physical problems |  |
| ([[9](#_ENREF_9), [18](#_ENREF_18), [24](#_ENREF_24), [26](#_ENREF_26), [29](#_ENREF_29), [44](#_ENREF_44)] | Worrying about threats to personal health |  |
| [[21](#_ENREF_21), [27](#_ENREF_27), [35](#_ENREF_35)] | The person's place of residence (city, village) |  |
| [[16](#_ENREF_16), [18](#_ENREF_18)] | Being busy and disrupting daily work |  |
| ([[3](#_ENREF_3), [10](#_ENREF_10)] | Lack of self-confidence |  |
| [[10](#_ENREF_10)] | Creating a false sense of self-management in patients and making decisions without consulting a doctor |  |
| [[4](#_ENREF_4)] | The feeling of being controlled |  |
| [[40](#_ENREF_40)] | Lack of previous use experience |  |
| [[24](#_ENREF_24)] | Inability to follow orders for the patient |  |
| ([[3](#_ENREF_3), [10](#_ENREF_10), [14](#_ENREF_14), [20](#_ENREF_20), [27](#_ENREF_27), [34](#_ENREF_34), [37](#_ENREF_37), [38](#_ENREF_38), [41](#_ENREF_41)] | Privacy concerns | Legal/Moral |
| [[14](#_ENREF_14)] | ethical challenges |  |

**Ref**.

1. Shaw RJ, Kaufman MA, Bosworth HB, Weiner BJ, Zullig LL, Lee SY, et al. Organizational factors associated with readiness to implement and translate a primary care based telemedicine behavioral program to improve blood pressure control: the HTN-IMPROVE study. Implementation science : IS. 201;8:106.

2. Robins LS, Jackson JE, Green BB, Korngiebel D, Force RW, Baldwin LM. Barriers and facilitators to evidence-based blood pressure control in community practice. Journal of the American Board of Family Medicine : JABFM. 2013;26(5):539-57.

3. Hanley J, Ure J, Pagliari C, Sheikh A, McKinstry B. Experiences of patients and professionals participating in the HITS home blood pressure telemonitoring trial: A qualitative study. BMJ open. 2013;3.

4. Flynn SJ, Ameling JM, Hill-Briggs F, Wolff JL, Bone LR, Levine DM, et al. Facilitators and barriers to hypertension self-management in urban African Americans: perspectives of patients and family members. Patient Prefer Adherence. 2013;7:741-9.

5. Patel S, Jacobus-Kantor L, Marshall L, Ritchie C, Kaplinski M, Khurana PS, et al. Mobilizing your medications: an automated medication reminder application for mobile phones and hypertension medication adherence in a high-risk urban population. Journal of diabetes science and technology. 2013;7(3):630-9.

6. McGillicuddy JW, Weiland AK, Frenzel RM, Mueller M, Brunner-Jackson BM, Taber DJ, et al. Patient attitudes toward mobile phone-based health monitoring: questionnaire study among kidney transplant recipients. J Med Internet Res. 2013;15(1):e6.

7. Lee K, Hoti K, Hughes JD, Emmerton L. Dr Google and the consumer: a qualitative study exploring the navigational needs and online health information-seeking behaviors of consumers with chronic health conditions. J Med Internet Res. 2014;16(12):e262.

8. Fiksdal AS, Kumbamu A, Jadhav AS, Cocos C, Nelsen LA, Pathak J, et al. Evaluating the process of online health information searching: a qualitative approach to exploring consumer perspectives. J Med Internet Res. 2014;16(10):e224.

9. Hall AK, Dodd V, Harris A, McArthur K, Dacso C, Colton LM. Heart failure patients' perceptions and use of technology to manage disease symptoms. Telemedicine journal and e-health : the official journal of the American Telemedicine Association. 2014;20(4):324-31.

10. Legido-Quigley H, Camacho Lopez PA, Balabanova D, Perel P, Lopez-Jaramillo P, Nieuwlaat R, et al. Patients' knowledge, attitudes, behaviour and health care experiences on the prevention, detection, management and control of hypertension in Colombia: a qualitative study. PloS one. 2015;10(4):e0122112.

11. Glynn L, Casey M, Walsh J, Hayes PS, Harte RP, Heaney D. Patients' views and experiences of technology based self-management tools for the treatment of hypertension in the community: A qualitative study. BMC Fam Pract. 2015;16:119.

12. Hallberg I, Ranerup A, Kjellgren K. Supporting the self-management of hypertension: Patients' experiences of using a mobile phone-based system. Journal of human hypertension. 2016;30(2):141-6.

13. Ganapathy R, Grewal A, Castleman JS. Remote monitoring of blood pressure to reduce the risk of preeclampsia related complications with an innovative use of mobile technology. Pregnancy Hypertension: An International Journal of Women's Cardiovascular Health. 2016;6.

14. Chung C-F, Munson S, Thompson M, Baldwin L-M, Kaplan J, Cline R, et al. Implementation of a New Kiosk Technology for Blood Pressure Management in a Family Medicine Clinic: from the WWAMI Region Practice and Research Network. The Journal of the American Board of Family Medicine. 2016;29:620-9.

15. Alnosayan N, Chatterjee S, Alluhaidan A, Lee E, Houston Feenstra L. Design and Usability of a Heart Failure mHealth System: A Pilot Study. JMIR human factors. 2017;4(1):e9.

16. Dou K, Yu P, Deng N, Liu F, Guan Y, Li Z, et al. Patients’ acceptance of smartphone health technology for chronic disease management: a theoretical model and empirical test. JMIR mHealth and uHealth. 2017;5(12):e7886.

17. Peters RM, Shivakumar N, Xu R, Javaherian K, Sink E, Patel K, et al. Assessing the Utility of a Novel SMS- and Phone-Based System for Blood Pressure Control in Hypertensive Patients: Feasibility Study. JMIR cardio. 2017;1(2):e2.

18. Rhoads SJ, Serrano CI, Lynch CE, Ounpraseuth ST, Gauss CH, Payakachat N, et al. Exploring Implementation of m-Health Monitoring in Postpartum Women with Hypertension. Telemedicine journal and e-health : the official journal of the American Telemedicine Association. 2017;23(10):833-41.

19. Haynes SC, Kim KK. A mobile system for the improvement of heart failure management: Evaluation of a prototype. AMIA Annual Symposium proceedings AMIA Symposium. 2017;2017:839-48.

20. Morrissey EC, Glynn LG, Casey M, Walsh JC, Molloy GJ. New self-management technologies for the treatment of hypertension: general practitioners' perspectives. Family practice. 2018;35(3):318-22.

21. Morton K, Dennison L, Bradbury K, Band RJ, May C, Raftery J, et al. Qualitative process study to explore the perceived burdens and benefits of a digital intervention for self-managing high blood pressure in Primary Care in the UK. BMJ Open. 2018;8(5):e020843.

22. Morrissey EC, Casey M, Glynn LG, Walsh JC, Molloy GJ. Smartphone apps for improving medication adherence in hypertension: patients’ perspectives. Patient preference and adherence. 2018;12:813.

23. Albrecht L, Wood PW, Fradette M, McAlister FA, Rabi D, Boulanger P, et al. Usability and acceptability of a home blood pressure telemonitoring device among community-dwelling senior citizens with hypertension: qualitative study. JMIR aging. 2018;1(2):e10975.

24. Portz JD, Bayliss EA, Bull S, Boxer RS, Bekelman DB, Gleason K, et al. Using the Technology Acceptance Model to Explore User Experience, Intent to Use, and Use Behavior of a Patient Portal Among Older Adults With Multiple Chronic Conditions: Descriptive Qualitative Study. J Med Internet Res. 2019;21(4):e11604.

25. Nelissen HE, Cremers AL, Okwor TJ, Kool S, van Leth F, Brewster L, et al. Pharmacy-based hypertension care employing mHealth in Lagos, Nigeria – a mixed methods feasibility study. BMC Health Services Research. 2018;18(1):934.

26. Chérrez-Ojeda I, Vanegas E, Felix M, Mata VL, Gavilanes AW, Chedraui P. Use and preferences of information and communication technologies in patients with hypertension: a cross-sectional study in Ecuador. Journal of multidisciplinary healthcare. 2019;12:583.

27. Xydopoulos G, Perry H, Sheehan E, Thilaganathan B, Fordham R, Khalil A. Home blood-pressure monitoring in a hypertensive pregnant population: cost-minimization study. Ultrasound in obstetrics & gynecology : the official journal of the International Society of Ultrasound in Obstetrics and Gynecology. 2019;53(4):496-502.

28. Xiao M, Lei X, Zhang F, Sun Z, Harris VC, Tang X, et al. Home Blood Pressure Monitoring by a Mobile-Based Model in Chongqing, China: A Feasibility Study. International journal of environmental research and public health. 2019;16(18).

29. Grant S, Hodgkinson J, Schwartz C, Bradburn P, Franssen M, Hobbs FR, et al. Using mHealth for the management of hypertension in UK primary care: an embedded qualitative study of the TASMINH4 randomised controlled trial. The British journal of general practice : the journal of the Royal College of General Practitioners. 2019;69(686):e612-e20.

30. Dugelay G, Kivits J, Desse L, Boivin JM. Implementation of home blood pressure monitoring among French GPs: A long and winding road. PloS one. 2019;14(9):e0220460.

31. Allen ME, Irizarry T, Einhorn J, Kamarck TW, Suffoletto BP, Burke LE, et al. SMS-facilitated home blood pressure monitoring: A qualitative analysis of resultant health behavior change. Patient education and counseling. 2019;102(12):2246-53.

32. Hoppe KK, Williams M, Thomas N, Zella JB, Drewry A, Kim K, et al. Telehealth with remote blood pressure monitoring for postpartum hypertension: A prospective single-cohort feasibility study. Pregnancy hypertension. 2019;15:171-6.

33. Barsky J, Hunter R, McAllister C, Yeates K, Campbell N, Liu P, et al. Analysis of the Implementation, User Perspectives, and Feedback From a Mobile Health Intervention for Individuals Living With Hypertension (DREAM-GLOBAL): Mixed Methods Study. JMIR Mhealth Uhealth. 2019;7(12):e12639.

34. Islam SMS, Cartledge S, Karmakar C, Rawstorn JC, Fraser SF, Chow C, et al. Validation and Acceptability of a Cuffless Wrist-Worn Wearable Blood Pressure Monitoring Device Among Users and Health Care Professionals: Mixed Methods Study. JMIR Mhealth Uhealth. 2019;7(10):e14706.

35. Silveira DV, Marcolino MS, Machado EL, Ferreira CG, Alkmim MBM, Resende ES, et al. Development and Evaluation of a Mobile Decision Support System for Hypertension Management in the Primary Care Setting in Brazil: Mixed-Methods Field Study on Usability, Feasibility, and Utility. JMIR Mhealth Uhealth. 2019;7(3):e9869.

36. Nichols M, Singh A, Sarfo FS, Treiber F, Tagge R, Jenkins C, et al. Post-intervention qualitative assessment of mobile health technology to manage hypertension among Ghanaian stroke survivors. Journal of the Neurological Sciences. 2019;406:116462.

37. Steinman L, Heang H, van Pelt M, Ide N, Cui H, Rao M, et al. Facilitators and Barriers to Chronic Disease Self-Management and Mobile Health Interventions for People Living With Diabetes and Hypertension in Cambodia: Qualitative Study. JMIR Mhealth Uhealth. 2020;8(4):e13536.

38. Cairns AE, Tucker KL, Crawford C, McManus RJ, Powell J. Implementing self-management: a mixed methods study of women's experiences of a postpartum hypertension intervention (SNAP-HT). Trials. 2020;21(1):508.

39. Jongsma KR, van den Heuvel JFM, Rake J, Bredenoord AL, Bekker MN. User Experiences With and Recommendations for Mobile Health Technology for Hypertensive Disorders of Pregnancy: Mixed Methods Study. JMIR Mhealth Uhealth. 2020;8(8):e17271.

40. van den Heuvel JFM, Ayubi S, Franx A, Bekker MN. Home-Based Monitoring and Telemonitoring of Complicated Pregnancies: Nationwide Cross-Sectional Survey of Current Practice in the Netherlands. JMIR Mhealth Uhealth. 2020;8(10):e18966.

41. Jahan Y, Rahman M, Faruque A, Chisti M, Kazawa K, Matsuyama R, et al. Awareness Development and Usage of Mobile Health Technology among Hypertensive Individuals in a Rural Community of Bangladesh: A Randomized Controlled Trial. Journal of Medical Internet Research. 2020;22.

42. Schoenthaler A, Leon M, Butler M, Steinhaeuser K, Wardzinski W. Development and Evaluation of a Tailored Mobile Health Intervention to Improve Medication Adherence in Black Patients With Uncontrolled Hypertension and Type 2 Diabetes: Pilot Randomized Feasibility Trial. JMIR Mhealth Uhealth. 2020;8(9):e17135.

43. Treskes RW, van Winden LAM, van Keulen N, van der Velde ET, Beeres SLMA, Atsma DE, et al. Effect of Smartphone-Enabled Health Monitoring Devices vs Regular Follow-up on Blood Pressure Control Among Patients After Myocardial Infarction: A Randomized Clinical Trial. JAMA Network Open. 2020;3(4):e202165-e.

44. Song T, Liu F, Deng N, Qian S, Cui T, Guan Y, et al. A Comprehensive 6A Framework for Improving Patient Self-Management of Hypertension Using mHealth Services: Qualitative Thematic Analysis. J Med Internet Res. 2021;23(6):e25522.

45. Walcott-Bryant A, Ogallo W, Remy SL, Tryon K, Shena W, Bosker-Kibacha M. Addressing Care Continuity and Quality Challenges in the Management of Hypertension: Case Study of the Private Health Care Sector in Kenya. J Med Internet Res. 2021;23(2):e18899.

46. Alessa T, Hawley M, Alsulamy N, Witte L. Using a commercially available app for self-management of hypertension: Acceptance and usability study in Saudi Arabia (Preprint). JMIR mHealth and uHealth. 2020;9.

47. Park S, Kum HC, Morrisey MA, Zheng Q, Lawley MA. Adherence to Telemonitoring Therapy for Medicaid Patients With Hypertension: Case Study. J Med Internet Res. 2021;23(9):e29018.

48. Bozorgi A, Hosseini H, Eftekhar H, Majdzadeh R, Yoonessi A, Ramezankhani A, et al. The effect of the mobile "blood pressure management application" on hypertension self-management enhancement: a randomized controlled trial. Trials. 2021;22(1):413.

49. Nau T, Owen A, Mazza D, Smith BJ. Engaging primary care providers in a mobile health strategy to support lifestyle change and blood pressure management. Digital health. 2021;7:20552076211066746.

50. Citoni B, Figliuzzi I, Presta V, Volpe M, Tocci G. Home Blood Pressure and Telemedicine: A Modern Approach for Managing Hypertension During and After COVID-19 Pandemic. High blood pressure & cardiovascular prevention : the official journal of the Italian Society of Hypertension. 2022;29(1):1-14.
